# Rapid, topology-based particle tracking for high-resolution measurements of large complex 3D motion fields

**DOI:** 10.1038/s41598-018-23488-y

**Published:** 2018-04-03

**Authors:** Mohak Patel, Susan E. Leggett, Alexander K. Landauer, Ian Y. Wong, Christian Franck

**Affiliations:** 1 School of Engineering, Brown University, Providence, RI 02912 USA; 2 Center for Biomedical Engineering, Brown University, Providence, RI 02912 USA; 3 Pathobiology Graduate Program, Brown University, Providence, RI 02912 USA

## Abstract

Spatiotemporal tracking of tracer particles or objects of interest can reveal localized behaviors in biological and physical systems. However, existing tracking algorithms are most effective for relatively low numbers of particles that undergo displacements smaller than their typical interparticle separation distance. Here, we demonstrate a single particle tracking algorithm to reconstruct large complex motion fields with large particle numbers, orders of magnitude larger than previously tractably resolvable, thus opening the door for attaining very high Nyquist spatial frequency motion recovery in the images. Our key innovations are feature vectors that encode nearest neighbor positions, a rigorous outlier removal scheme, and an iterative deformation warping scheme. We test this technique for its accuracy and computational efficacy using synthetically and experimentally generated 3D particle images, including non-affine deformation fields in soft materials, complex fluid flows, and cell-generated deformations. We augment this algorithm with additional particle information (e.g., color, size, or shape) to further enhance tracking accuracy for high gradient and large displacement fields. These applications demonstrate that this versatile technique can rapidly track unprecedented numbers of particles to resolve large and complex motion fields in 2D and 3D images, particularly when spatial correlations exist.

## Introduction

Comprehensive tracking of tracer particle trajectories can elucidate complex behaviors in soft materials, fluid flows, and biological motion by resolving local inhomogeneities in space and time. For instance, the displacement of fiducial markers embedded within a solid or fluid medium can reveal local microrheological properties^1–7^, cell-generated deformations within a soft biomaterial^8–18^, or turbulent flows utilized for biological propulsion^19–21^. Moreover, directly tracking biological objects of interest such as single molecules^22–24^, viruses and bacteria^25–29^, and motile cells^30–33^ can reveal collective phenomena and heterogeneous phenotypes. However, particle tracking is computationally expensive and requires careful optimization of imaging conditions and thus remains highly challenging. The unambiguous resolution of particle motion typically requires displacements smaller than half of the interparticle spacing, often necessitating frequent time-lapse imaging^34,35^. Consequently, existing algorithms are inefficient when tracking large numbers of particles (tens of thousands or more) undergoing large motion over an extended duration or many image frames. New tracking algorithms exhibiting ultrahigh efficiency and accuracy based on computer vision techniques are necessary for extreme motion fields in physical and biological systems.

Single particle tracking (SPT) methods are typically based on a two step process: 1) particle positions are identified at each time frame, then 2) particle positions are linked together into trajectories over consecutive frames. Particles can be reliably localized with sub-pixel accuracy in both 2D and 3D images using Gaussian fitting methods^36–38^ based either on a nonlinear least squared criterion or maximum likelihood estimator, or the radial symmetry method^39,40^. Once located, the particles can be tracked using approaches including nearest neighbor search, relaxation methods^41,42^, feature vector based methods^12,34^, and more classical minimization of a specific motion cost function^43,44^ using the sum of squared distances across time points. These methods accurately track particles subjected to small motion fields. However, tracking large particle motions presents a significant challenge due to the uncertainty of assigning new positions to identical particles. Moreover, they are not computationally efficient when tracking large numbers of particles undergoing large amplitude motion fields. Although some application-specific algorithms can capture large displacements^45^, these methods require inference of a priori knowledge of the underpinning physics behind the motion and thus are not readily adaptable to general motion fields of unknown character.

Complex motion fields can also be measured using correlation-based methods like digital image correlation (DIC) or particle image velocimetry (PIV), which do not require explicit information about particle positions. These correlation-based image subset matching methods are widely used^46,47^, but the inherent averaging nature of subset-matching schemes smooths out high frequency information^48–50^, and are computationally expensive since mathematical operations are applied across the entire 2D image or 3D volume^51,52^. Instead, SPT reconstructs a complex deformation field based on the spatially discretized motion of tracer particles. To resolve high spatial frequency information, high particle seeding densities are necessary. According to the Nyquist criterion, SPT methods have a theoretical spatial frequency recovery limit with the smallest resolvable wavelength twice the interparticle separation distance^11^. Increasing the overall particle seeding density reduces the interparticle separation, which then limits the relative displacements that can be accurately and efficiently resolved using existing algorithms. This establishes a tradeoff whereby higher particle densities permit high spatial resolution but lower particle densities are needed to resolve large displacements. These issues are expected to worsen as camera sensors increase in size, allowing greater numbers of particles within a given field of view.

Here, we present a new particle tracking scheme called Topology-based Particle Tracking (T-PT) to address the challenges of current SPT methods in reconstructing complex, large motion fields with high computational efficiency and spatial resolution. Our approach utilizes feature vectors that encode nearest neighbor positions, a rigorous outlier removal scheme, and an iterative deformation warping scheme^51^. T-PT is designed to perform optimally for motion fields that exhibit some spatial correlations, although it can also be employed to track purely random motions. We demonstrate that our general purpose method accurately tracks a large number of particles undergoing large and high spatial gradient displacements with high computational efficiency. By including descriptive, multi-attribute information about the particles, e.g. particle color, size or shape, in addition to their geometric position, we show that the displacement resolution of our technique can be significantly enhanced for more accurate detection of large motion fields. Furthermore, we characterize the performance and versatility of our algorithm using a combination of simulated and experimental images, including non-affine deformations of soft materials, complex fluid flows, and cell-generated substrate deformations.

## Results

### Particle Tracking Using Feature Vectors with Relative Neighbor Positions

**Figure 1:**
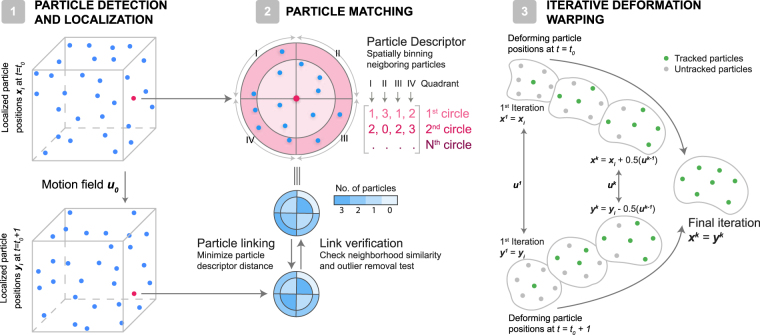
Schematic of the T-PT algorithm. Step 1: Particles in the images are detected and their centers localized with subpixel accuracy using the radial symmetry method^39,40^. Step 2: T-PT is a feature-vector-based particle method. Particles are linked via a unique “particle descriptor” or feature-vector, which is created by spatially binning the neighboring particles. The particles are linked one-to-one between consecutive image frames by minimizing the particle descriptor *L*^2^ norm distance. These particle links are temporary and are subjected to outlier removal schemes^53^ to eliminate potential false particle links. The links verified by the outlier removal scheme are converted to actual particle matches. Step 3: Iterative deformation warping^51^ is used to resolve large particle displacements. Here, the positions of particles in the reference and deformed frames are iteratively warped by a linearized displacement field computed from matched particles, until the positions of the particles in the two consecutive frames coincide. The whole process of particle matching and iterative deformation warping is performed iteratively until a set of convergence criteria is met.

Topology-based Particle Tracking (T-PT) utilizes feature vectors in a three-step algorithm (Fig. 1). First, particles centers are localized. Second, particles are linked between consecutive image frames using a new particle descriptor (feature vector), which encodes the spatial positions of their nearest neighbor particles for each time frame. Third, particles in consecutive time frames are compared using an iterative warping scheme to track large particle motions in time^51^. These three steps will be described in greater detail using a conceptual example of particles displaced by a 3D motion field.

The first step in most tracking schemes is the detection and localization of particle centers within each image (Fig. 1). We utilize image thresholding based on a user-specified cutoff to segment particle voxels from background voxels in the images. The particle centroids are then rapidly localized with sub-voxel accuracy using the radial symmetry method^39,40^.

The second step associates the particle positions in the reference (*t* = *t*_*o*_) and deformed volumes (*t* = *t*_*o*_ + 1) with a “particle descriptor” or feature vector, based on the relative spatial positions of their nearest-neighbor particles (Fig. 1). This step defines a unique local particle signature based on the topological arrangement of randomly located neighbors, which aids in linking consecutive particle positions in time. For each particle, the positions of *n* nearest neighbor particles are stored. A spherical shell with a radius equal to the distance of the farthest particle from the particle of interest is then created around each particle. This spherical shell is further divided into *k* concentric shells of equal volume. Each of the concentric shells is further split into eight octants using the basis vectors in the reference volume, dividing the space around the particle of interest into 8*k* bins. The relative positions of the *n* neighboring particles is encoded into the particle descriptor by binning these particles into the 8*k* bins. The design of this feature descriptor is advantageous since it encodes the *relative* spatial positions of neighboring particles. This allows these particles sufficient freedom to rearrange during deformation while retaining a similar feature descriptor, since particles tend to remain within the same bin.

Particle positions are linked together in time by partitioning the reference (*t* = *t*_*o*_) and the deformed (*t* = *t*_*o*_ + 1) volumes into subsets. The particles are linked one-to-one from the reference image subset to the corresponding deformed image subset such that a chosen particle pair has the minimum *L*^2^ particle descriptor distance. The algorithm enforces one-to-one particle linking conditions by discarding any particle links which are not bijective. An outlier removal procedure on particle links then eliminates potential false links. We utilize the neighborhood similarity test, which evaluates whether a particle linkage is plausible based on whether at least *p* of *q* neighbors are conserved between the reference and deformed volumes, in addition to the universal median test^53^, which removes potential outliers by eliminating particle displacements above a user-defined value based on the normalized residual of the median neighbor particle displacement. The links verified by these two tests are stored as successful particle matches.

The third step in our algorithm uses an iterative deformation warping (IDM) scheme to accurately and efficiently track large and complex particle displacements^51^. IDM resolves any general nonlinear deformation by iteratively warping the reference and deformed images using a linearized displacement field until they converge to the same final configuration. The technique is adapted to deform particle positions in the reference and deformed images until the particle positions coincide. In addition to improving the large particle displacement recovery process, IDM also improves the similarity of particle descriptors in the reference and deformed volumes, further enhancing the accuracy of particle linking. The whole process of the particle matching and deformation warping is iteratively performed on decreasing subset sizes from 256 to 16 voxels to reduce the overall search region until the convergence criteria is fulfilled. The detailed description of various steps in the algorithm can be found in the Methods section and Supplementary Note 7.

### Tracking Accuracy and Efficiency in Resolving Complex Motion Fields

The performance of our T-PT algorithm was evaluated by synthetically generating a 3D volume of 512 × 512 × 192 voxels, with a signal-to-noise ratio (SNr) of 25, that contained 50,000 randomly distributed, identically-sized spherical particles with a particle density of 9.94 × 10^−4^ particles/voxel^3^ (Supplementary Figure 1). These voxel intensities were further sampled from a Poisson distribution in order to simulate shot noise during experimental image acquisition.

**Figure 2:**
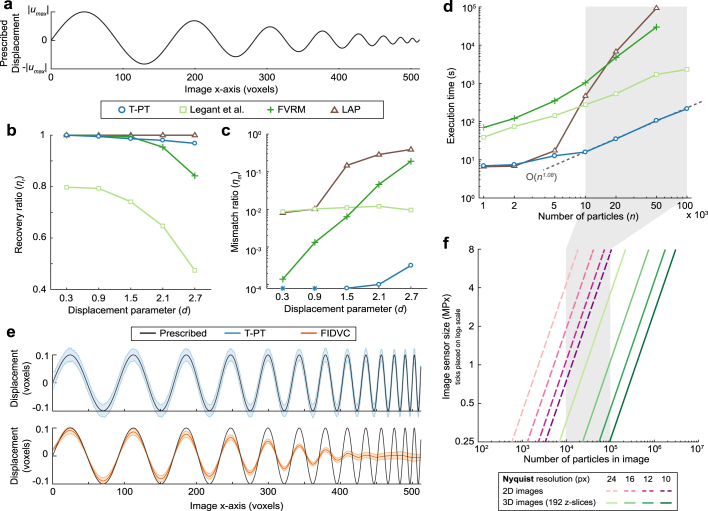
Performance characterization of the T-PT technique. (**a)** Analytically prescribed displacement field used for assessing the performance of our T-PT technique. The prescribed displacement field is composed of a sinusoidal field of linearly decreasing spatial wavelength from 200 to 10 pixels. The displacement parameter (*d*) governs the |*u*_*max*_| of the displacement field. (**b)** Plot of the recovery ratio of various algorithms versus increasing displacement parameter, *d*. (**c)** Plot showing the mismatch ratio of different algorithms against increasing displacement parameter, *d*. The mismatch ratio for T-PT for *d* = 0.3 and 0.9 is zero. Since a value of zero is not defined in a log plot, it is indicated by blue stars in the plot. (**d)** Plot illustrating the execution time of each algorithm against synthetic images seeded with various numbers of particles. The execution time for FVRM and TrackMate for 100,000 particles is not shown in the plot, as the algorithm execution was terminated when its execution time exceeded 10^5^ seconds. (**b**,**c**,**d)** ‘T-PT’ indicates the topology-based particle tracking method introduced here; ‘Legant *et al*.’ indicates the feature-vector based particle tracking method by Legant *et al*.;^12,15^ ‘FVRM’ indicates the feature-vector based relaxation method;^34^ ‘LAP’ indicates Jaqaman *et al*.’s LAP-based algorithm^24^ implemented in TrackMate^54^. (**e)** Plot comparing the ability of T-PT and FIDVC^51^ to recover high spatial frequency motion content. The shaded region indicates the standard deviation of the recovered displacement magnitude. (**f)** Number of particles required in 2D and 3D images (192 *z*-slices) to resolve the imposed displacement field at the spatial Nyquist resolution versus camera detector resolution in megapixels.

To generate the deformed volume, the particles were then displaced by a sinusoidal field of linearly decreasing amplitude and spatial wavelength from 200 to 10 voxels (Fig. 2a). The complexity of the displacement field arises because it has both a region of large displacement and high spatial frequency motion. To quantify the displacement magnitude between an image pair, the displacement parameter *d* is defined as the ratio of maximum displacement magnitude |*u*_*max*_| to mean interparticle separation distance *r*_*o*_, i.e., $d=\frac{|{u}_{max}|}{{r}_{o}}$. For the particle seeding density shown in the images, the particle separation distance is *r*_*o*_ = 6.217 voxels. For “small” displacements of *d* < 0.5, a nearest neighbor search will be sufficient to track all particles, assuming a uniform volumetric particle distribution. However, accurate particle tracking becomes challenging for *d* > 0.5, as the true particle link is not necessarily the nearest neighboring particle in the local neighbor search. Thus, we evaluated the performance of this algorithm and other current SPT techniques as a function of increasing *d* for the prescribed displacement field (Fig. 2a). In particular, we considered three available methods, Legant *et al*.’s feature-vector based tracking method^12,15^, Jaqaman *et al*.’s linear assignment problem (LAP)-based algorithm^24^ implemented in TrackMate^54^, and a feature-vector-based relaxation method (FVRM)^34^.

The algorithm performance was evaluated using positive and false positive linkages as the displacement parameter (*d*) was systematically varied. The recovery ratio *η*_*r*_ is defined as the ratio of the total number of detected particle links found by the algorithm to the total number of existing particle links in between an image pair (Fig. 2b). For *d* < 1.5, T-PT, FVRM, and TrackMate exhibit very high recovery ratios *η*_*r*_ ~ 1. As *d* increases further, the recovery ratio remains nearly unity for T-PT and Trackmate out to *d* ~ 2.7, while FVRM decreases to *η*_*r*_ ~ 0.9. Legant *et al*.’s method has the lowest *η*_*r*_, which also decreases with increasing *d*. The mismatch ratio *η*_*m*_ is the ratio of false particle links found by the algorithm to the total number of particle links detected by the algorithm. T-PT does not find any false-positive links *η*_*m*_ ~ 0 for *d* < 1.5, but increases to a maximum of *η*_*m*_ ~ 6 × 10^−3^ for *d* = 2.7. For FVRM, *η*_*m*_ ~ 9 × 10^−3^ for *d* = 0.3, which increases roughly exponentially up to *η*_*m*_ ~10^−1^ for *d* = 2.7. Finally, Legant *et al*.’s method remains roughly constant at *η*_*m*_ ~ 10^−2^ over this range of *d*, while TrackMate increases from *η*_*m*_ ~ 10^−2^ at *d* = 0.3 to *η*_*m*_ ~ 0.6 at *d* = 2.7. Overall, these metrics indicate that T-PT enables accurate tracking at high particle densities even for motion fields with very large displacement information.

In addition to tracking large deformations, the number of particles that can be efficiently tracked must be considered. Tracking a significant number of particles (~10^4^) undergoing large deformations, i.e., *d* > 0.5 can be prohibitively expensive, particularly for large numbers of time-lapse images. The computational efficiency as a function of the total number of tracked particles was evaluated using the same displacement field as before (Fig. 2a, *d* = 2.1). For all algorithms, the execution time is small for low particle densities and increases significantly with the number of particles (Fig. 2d). For high seeding densities, our T-PT has the fastest execution time, tracking 10^5^ particles in 215.3 seconds. This is approximately 10 times faster than Legant *et al*.’s method, which is the second fastest method. As shown in Fig. 2d, for the given motion field our T-PT’s execution time approximately scales as *O*(*n*^1.08^), where *n* is the number of particles in the images.

At present and to the best of our knowledge, the only motion tracking techniques that perform similarly for large particle numbers are correlation-based methods. However, correlation-based methods tend to underresolve high spatial frequency motion content due to the intrinsic averaging nature of their subset-based image matching routines. To show this effect, we considered a sinusoidal displacement field with a magnitude of 0.1 voxels with a linearly decreasing spatial frequency from 100 to 10 voxels. This displacement field is prescribed to an image of size 512 × 512 × 192 voxels embedded with 50,000 particles and a SNr of 25. We compare the prescribed displacement field against the high spatial frequency displacement content recovered by T-PT and fast iterative digital volume correlation (FIDVC)^51^, a correlation based method (Fig. 2e). FIDVC progressively underpredicts the true displacement magnitude with increasing spatial frequency and fails to recover the highest spatial frequency displacement content. In comparison, T-PT recovers the high spatial frequency motion but has a larger standard deviation in its resolved motion field than FIDVC, since T-PT does not any employ intrinsic spatial averaging or noise suppression calculations. T-PT also showed a significant speed improvement over the correlation based FIDVC method, as T-PT and FIDVC measured the displacements in 45.2 seconds and 391.3 seconds, respectively.

Recent advancements in camera sensor technology have enabled larger fields of view, allowing more particles to be imaged per field of view. For instance, EMCCD cameras typically have sensor sizes of 512 × 512 pixels (0.26 MPx) or better. The first-order estimate for the Nyquist spatial resolution of the recovered motion field is twice the mean interparticle spacing. To resolve a motion field at Nyquist spatial resolution of 16 pixels, corresponding to a resolution of a single particle by four pixels in each dimension, approximately ~10^3^ particles must be tracked for a single 512 × 512 pixel 2D image, but ~2 × 10^4^ particles must be tracked in a 512 × 512 × 192 voxel 3D volume (Fig. 2f; details in Supplementary Note 2). Existing particle tracking algorithms exhibit adequate performance for 10^4^ particles (Fig. 2d). Yet recent developments in sCMOS camera technology with sensor sizes of 2048 × 2048 pixels (4.2 MPx), corresponding to tracking 3 × 10^5^ particles per 3D volume (2048 × 2048 × 192 voxels), which becomes impractical for existing algorithms (Fig. 2f). Future research may use even greater camera resolution, requiring larger numbers of particles to be tracked, perhaps 10^6^ or more. The efficient performance of this algorithm for large displacements and high particle numbers is thus highly promising for emerging imaging technologies. Moreover, it should be noted that this algorithm is parallelized and parallel computing could be utilized to further reduce execution time, if needed.

We have also compared the performance of T-PT to resolve random particle motion against other SPT algorithms (Supplementary Note 5). TrackMate, followed by T-PT, had the most accurate tracking performance. The execution time of T-PT was at least an order of magnitude faster than the second fastest method, and T-PT and TrackMate had similar particle tracking accuracy for small random particle motion (*d* < 0.25). Thus, T-PT is preferable for applications involving a high number of particles undergoing small random motion. In addition, we evaluated the performance of T-PT for time-lapse data where particles randomly appeared and disappeared between image frames, which simulates experiments where particles merge and split (Supplementary Note 6). T-PT achieved a *η*_*r*_ ~ 0.98 and *η*_*m*_ ~ 7 × 10^−5^ between image frame 0 and image frame 5 for 10% of particles randomly seeded and removed in each image frame. T-PT showed that it could accurately track particles over time-lapse images even when a large fraction of particles appeared and disappeared between image frames.

### Large Deformation Tracking in Non-Affine Soft Materials and Fluid Flows

**Figure 3:**
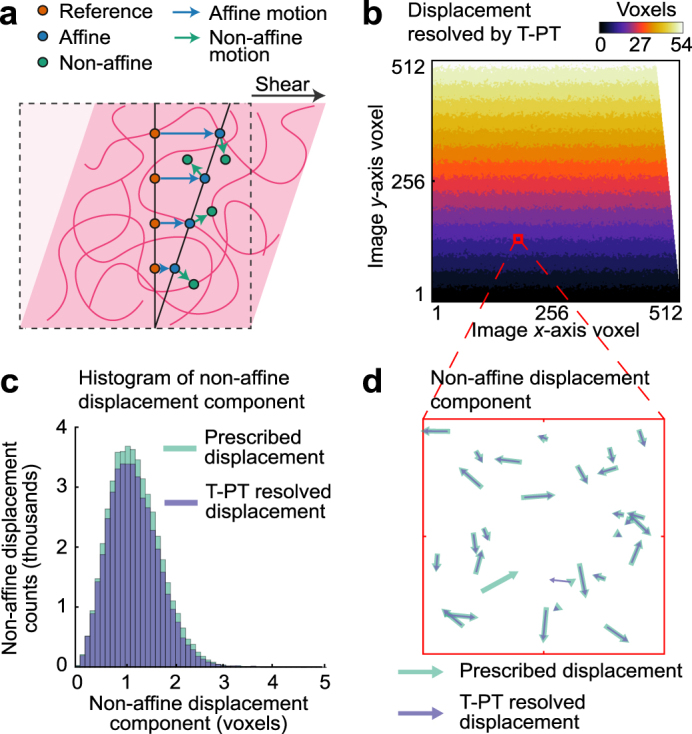
Application of T-PT in resolving non-affine shear deformations in the rheology of soft materials. (**a)** Schematic of the analytically prescribed affine and non-affine displacement fields as seen in many soft materials^55^. (**b)** Contour plot of the displacement magnitude recovered by T-PT from the synthetic images. (**c)** Histogram of the applied and recovered non-affine displacement component of the prescribed motion field in **a**. (**d)** Example comparison of the non-affine displacement component recovered by T-PT against the prescribed non-affine displacement applied to the particles in the synthetic images. The arrows in the figure indicate the direction and relative magnitude of the 3D displacement projected on the *x*-*y* plane.

**Figure 4:**
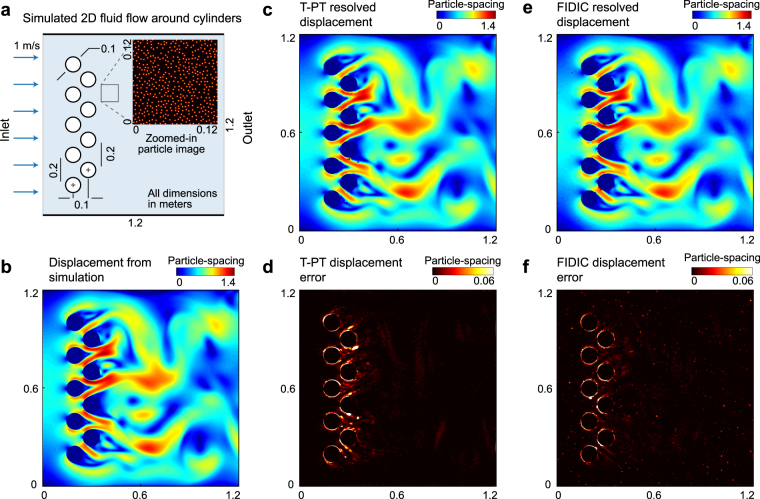
Application of T-PT in resolving a complex fluid flow across a stack of cylinders. (**a)** Schematic of a 2D Computational Fluid Dynamics (CFD) flow simulation around the periodically spaced cylinders. This displacement field is prescribed to a synthetically generated particle image pair. Inset shows a zoomed-in particle image subset to highlight the high particle seeding density in the images. (**b)** Contour plot of the magnitude of the 2D displacement field extracted from the CFD simulation. (**c**,**e)** Contour plot of the displacement vector magnitude recovered by T-PT and FIDIC, respectively from the synthetic images. (**d**,**f)** Contour plot of the displacement error from the recovered displacement field via T-PT and FIDIC, respectively.

To highlight the versatility of our T-PT algorithm, we chose two examples of potential interest to the biological and physical sciences communities. The first example examines the reconstruction capability of our technique to accurately capture both affine and non-affine motion fields in an idealized soft material undergoing a nominally applied homogeneous shear deformation (Fig. 3a–d). The second example investigates the resolution capability of T-PT to recover a complex, interacting fluid flow field across a stack of periodically-spaced cylinders at Reynolds number *Re* = 10,000 (Fig. 4a–f). These examples are simulated using synthetically produced images from analytically (example 1) and computationally (example 2) derived motion fields.

Particle tracking microrheology in soft materials enables resolution of non-affine deformations due to inhomogeneities in network architecture or enthalpic deformations of structural components (i.e. semiflexible or rigid polymers), which cannot be elucidated using bulk rheology^55^. We synthetically generate a 3D image set of 512 × 512 × 192 voxels seeded with 50,000 particles undergoing a nominal shear (*γ*) deformation of 10%. Onto this homogeneous deformation, we superimpose random displacement vectors to create a non-affine displacement field as might occur in fibrous materials such as collagen or fibrin (Fig. 3a). The regularly spaced bands in displacement contours measured by T-PT (Fig. 3b) indicate that it recovered the affine, finite (*γ* = 0.1) homogeneous shear displacement field accurately. The uneven and jagged border between the bands arises from the non-affinity in particle displacements. The 3D image pair was generated with a SNr of 25 and T-PT reconstructed the displacement field with a mean absolute displacement error of 0.145 ± 0.825 voxels. Concurrently, T-PT resolved most of the non-affine displacement components of the prescribed displacement field, as seen in the histogram of the superimposed non-affine displacement in Fig. 3c. Similarly, Fig. 3d visualizes the randomness in the prescribed non-affine displacement field in a subset of the 3D images, showing that T-PT resolves most of the non-affine displacement components. T-PT tracked the particles in the images with a recovery ratio of 0.922 and a mismatch ratio of 0.0184, indicating high accuracy in resolving the prescribed non-affine shear motion field. T-PT maintained similar accuracy across various applied shear deformations as the extent of non-affinity was varied (Supplementary Note 4).

Experimental fluid dynamics is another research area that commonly employs various SPT techniques to reconstruct complex velocity fields by tracking fiducial particles within the fluid flow. Examples include measuring fluid flow fields around bats^19^, jellyfish^20^, or bacteria^29^. The motion fields are usually recovered either by particle image velocimetry (PIV) or particle tracking velocimetry (PTV) methods. As a correlation based technique, PIV has limited capability in capturing high spatial gradient flow fields^56^ (Fig. 2e). PTV can improve the spatial resolution of the measured flow field^56,57^, but two-frame PTV techniques are limited to small particle displacements^42^. PTV techniques usually require multi-frame information to resolve large particle displacements^57^, which imposes experimental challenges.

To show T-PT’s ability to recover large and high spatial gradient displacement data encountered in an unsteady, complex flow field between two image frames, we simulate a 2D flow around a stack of periodically spaced cylinders in COMSOL (Fig. 4a). The numerical simulation details are described in the Methods section. The flow passing between the cylinders creates a complex motion field of varying spatial frequencies, with large displacements (peak value of 1.42 times the mean particle-spacing) as well as steep gradients (peak value of 2.9) (Fig. 4b). At a particular time, the displacement field is extracted from the simulation and prescribed to a synthetic 2D particle image pair. The displacement field is then reconstructed from the particle motion using T-PT and FIDIC^51^ (Fig. 4c,e) and compared with the original displacement field (Fig. 4d,f). The errors for T-PT are mostly localized around the cylinder borders mainly because of scattered data interpolation scheme limitations. The error contribution in the recovered displacement field from the particle mismatch is relatively small as the mismatch ratio was 1.33 × 10^−4^. T-PT and FIDIC resolve the displacement field with a mean absolute error of 0.0325 ± 0.147 pixels and 0.0387 ± 0.133 pixels, respectively. The particles were tracked with a recovery ratio of 0.965, showing T-PT’s ability to accurately resolve the complex displacement field imposed. The computational times for T-PT and FIDIC to resolve the displacements were 76.1 seconds and 81.6 seconds, respectively.

### Improved Tracking Accuracy Using Multi-Attribute Particles

**Figure 5:**
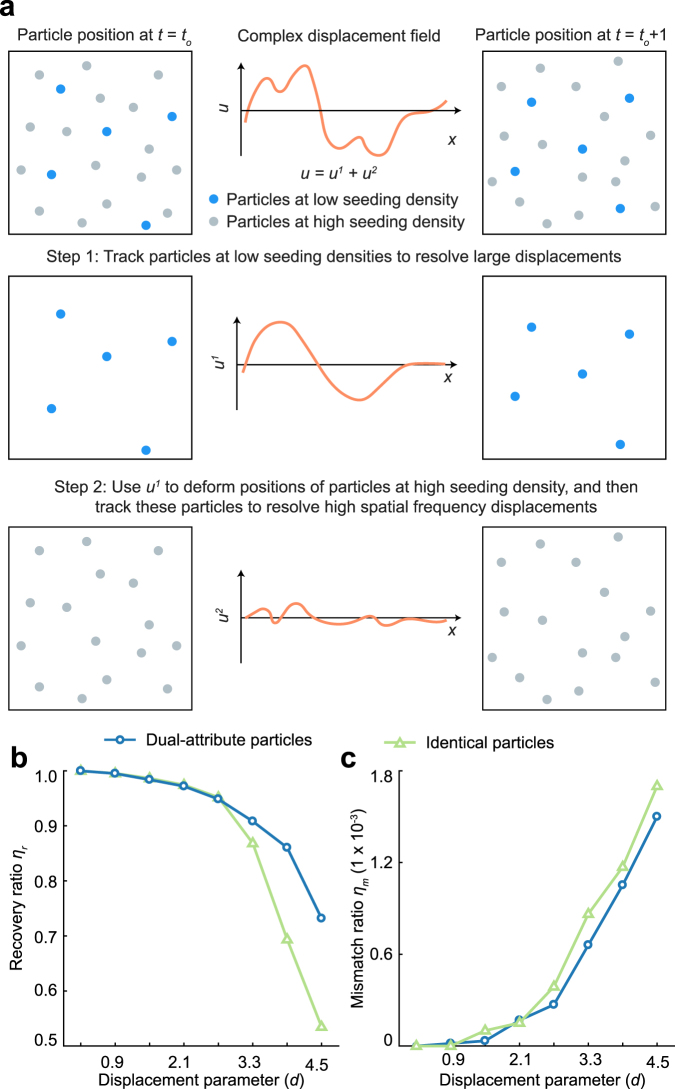
(**a)** Schematic overview of tracking multi-attribute particles. To resolve a complex displacement field, the images are seeded with two different types of particles, differentiated either by color, shape or size as an example. The use of multi-attribute particles allows a particle tracking algorithm to more accurately resolve a complex motion field composed of both large displacements and high spatial frequency motion content. (**b)** Plot comparing the recovery ratio of our T-PT results using single (i.e., identical) attribute vs. dual-attribute particles over a range of displacement parameters. (**c)** Mismatch ratio of T-PT results using single-attribute vs. dual-attribute particles over a range of displacement parameters.

Accurate reconstruction of complex, high spatial frequency displacement content requires high particle seeding densities to reduce the Nyquist limit. However, increasing particle seeding density reduces the interparticle separation distance, and thus challenges the ability of state-of-the-art tracking algorithms, including our own, to resolve very large deformations. Thus, a tradeoff exists between resolving large deformations and recovering high spatial frequency displacement information. While T-PT can accurately track large displacements, its recovery ratio, as with other SPT techniques, decreases dramatically as the imposed displacements become too large. To improve the tracking performance of our T-PT algorithm for these larger displacements, we enhanced T-PT by taking advantage of any additional particle information available beyond spatial positioning. For example, such multi-attribute information can include the particle fluorescent emission wavelength (color), size or shape, et cetera. To benchmark the performance increase of utilizing multi-attribute particle information we synthetically generated a volumetric image pair of size 512 × 512 × 192 voxels seeded with 60,000 identical particles. Next, we augment the monodispersed solution of particles by prescribing one additional distinguishing attribute to a 10,000 particles subset and another attribute to the remaining 50,000 particles, creating two particle groups with low and high seeding densities (Fig. 5a). Experimentally, as we show later, particles of different colors or different sizes are used. The dual-attribute synthetic particle images are prescribed the original displacement field from Fig. 2a. The particles at low seeding density have larger interparticle spacing, and thus will provide higher accuracy in resolving large displacements at low spatial frequency, whereas the high seeding density particles with small interparticle spacing provide the best estimate in the high spatial frequency content recovery process. We first track the particles at low seeding densities to resolve large deformations with low spatial frequency content in the applied motion field. Then, we use the iterative deformation warping scheme^51^ to deform the positions of all particles, including the ones at the high seeding densities according to this field. Finally, particles at the high densities are accurately tracked to recover the high spatial frequency and displacement gradient content. Multi-color particles have been previously used to increase the spatial resolution of the motion field measurement^11^. Now, our approach uses multi-attribute particles to resolve large displacement content at high spatial resolution, and thus provides a more general methodology for including any distinguishing particle characteristics in addition to color and position.

The inclusion of a single additional attribute improves the tracking capability of the T-PT algorithm significantly, with the most improvement seen in the recovery ratio, *η*_*r*_ (Fig. 5b). For *d* < 2, *η*_*r*_ is comparable for identical and dual-attribute particle tracking. Nevertheless, for *d* > 3, the demonstrated dual-attribute particle tracking permits higher recovery ratios, reaching *η*_*r*_ = 0.75 at *d* = 4.5, roughly 50% better than monodispersed particles. The mismatch ratio *η*_*m*_ is roughly consistent for dual-attribute and identical particle tracking with varying *d* (Fig. 5c). The details of the outlier removal scheme explains this trend. This step removes false particle matches, hence controlling the mismatch ratio. Since the outlier removal scheme is unmodified between identical particle and dual-attribute cases, both have similar mismatch ratios. This conceptual example uses color as a multi-attribute property, but the feature descriptor can straightforwardly be used with any other particle property.

### Improved Resolution of Cell-Generated Substrate Deformations

**Figure 6:**
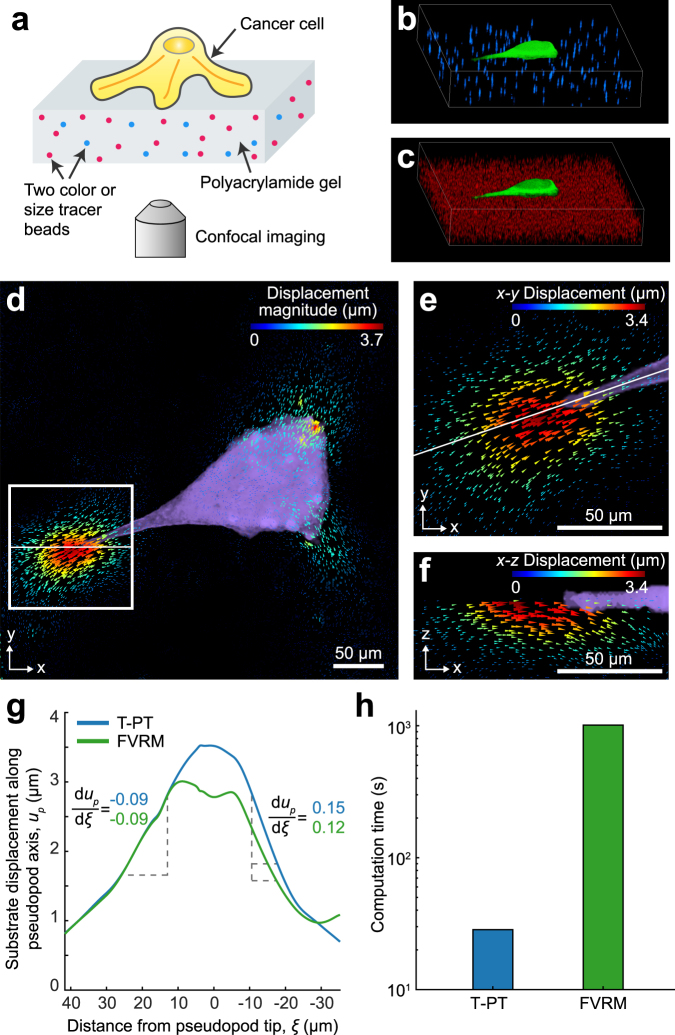
Three-dimensional application of T-PT for measuring cell-induced substrate deformations. (**a)** Schematic of the experimental setup to measure substrate deformations applied by a breast cancer cell adherent to a soft polyacrylamide (PA) gel substrate functionalized with collagen I. The cell is fluorescently labeled with cytoplasmic green-fluorescent protein, and the PA gel is embedded with two different sized and fluorescently labeled microspheres. (**b)** Volumetric confocal image of 2 μm blue microspheres seeded at a low particle density in the PA gel, with the cell shown in green. (**c)** Volumetric confocal image of 1 μm red microspheres seeded at a high particle seeding density in the PA gel, with the cell shown in green. (**d)** 3D displacement magnitude of the extracellular matrix deformations recovered by T-PT by tracking the dual-attribute particles. (**e)** Inset outlined in **d** magnified to show the *x*-*y* displacement component. (**f)** Magnified cross-sectional view of the inset outlined in **d** to show the *x*-*z* displacement component. (**g)** Displacement component magnitude measured by T-PT and FVRM algorithms along the pseudopod axis highlighted by the white line in **e**. (**h)** Comparison of the computation time for T-PT and FVRM for recovering the 3D displacement information in the volumetric image pair of size 512 × 512 × 101 voxels.

Finally, we analyze experimental images of cell-generated deformations using our T-PT algorithm. Adherent mammalian cells use focal adhesions to apply highly localized forces to soft biomaterial substrates, causing displacements with large magnitudes and steep gradients^16,58,59^. To reconstruct this complex motion field accurately, particle tracking algorithms capable of high spatial resolution are required^60^. Our experimental system consists of a fluorescently-labeled breast cancer cell (MDA-MB-231, metastatic breast adenocarcinoma stably transfected with a green fluorescent protein) adherent to a collagen-I conjugated soft polyacrylamide (PA) gel (stiffness ≈1.5 kPa) (Fig. 6a). Two sets of polystyrene microspheres with distinct size and fluorescent label are embedded within the PA gel. 2 μm blue tracer particles are injected at a low seeding density of 1.01 × 10^−5^ particles/voxel (Fig. 6b) and 1 μm red particles are injected at a high seeding density of 6.38 × 10^−4^ particles/voxel (Fig. 6c). The dual-attribute particles, as described earlier, help T-PT to recover both the large and high gradient displacement content. A 3D volumetric image pair of size 512 × 512 × 101 voxels was recorded via a standard confocal microscope (see Methods), and our 3D T-PT algorithm tracked the particles in the images to recover the displacement within the PA substrate.

The breast cancer cell exhibited an elongated, spindle-like morphology representative of a mesenchymal morphology in sparsely plated culture conditions^61^. In particular, near the trailing edge (Fig. 6d, left), the adhesive contact is associated with a relatively sharp spatial gradient deformation ($|{\rm{\partial }}{u}_{p}/{\rm{\partial }}\xi |=0.15$, where *ξ* is the distance to the pseudopod tip) (Fig. 6e,f). These localized large deformations are typically observed near adhesive and focal contacts in various adherent cell types^16,58,59^. The T-PT surface displacement field along the cell body can be compared with the feature-vector based relaxation method (FVRM)^34^, another state-of-the-art SPT technique used to measure cell-generated deformations^18^. In general, both methods yield similar displacement field estimates. However, in comparison to T-PT, FVRM under-predicted the peak displacement magnitude by 15% (Fig. 6g). Behind the trailing edge along the cell body axis (*ξ* ~ 40), both T-PT and FVRM yield comparable displacement magnitudes of ~1 μm at a displacement gradient magnitude of 0.09. Near the pseudopod, at *ξ* = 0, FVRM underestimates the magnitude of the displacement field by ~1 μm relative to T-PT. Moreover, ahead of the trailing edge at *ξ* < 0, T-PT resolved a higher gradient magnitude of 0.15 compared to 0.12 using FVRM. These results indicate that T-PT better recovers large deformations and high gradient displacements. T-PT recovered the displacement field with an SNr of 48. We calculated the displacement SNr as the ratio of peak particle displacement magnitude (3.7) to the noise floor of the particle displacement (0.077). Furthermore, T-PT tracked 11,247 beads inside the image pair in 27.67 seconds, which is approximately 15 times faster than FVRM (Fig. 6h). The accuracy and efficiency of tracking particles with T-PT is promising for traction force microscopy applications, among others. The accuracy and computational efficiency advancement of our T-PT method will become more pronounced with higher native image resolution and higher particle seeding densities for resolving motion fields at very high Nyquist spatial resolution (Fig. 1e,g).

## Discussion

T-PT is a new feature-vector-based particle tracking algorithm for large displacements and particle numbers that uses feature descriptors that encode the relative spatial positions of neighboring particles. This approach improves on existing tracking algorithms by incorporating this newly developed feature descriptor, rigorous outlier removal schemes, and an iterative deformation warping method^51^. This algorithm was evaluated computationally using a frequency-varying sinusoidal displacement field, a model soft material with affine and non-affine deformations, and a spatially heterogeneous fluid flow through a periodic array of cylinders. This algorithm was applied to reconstruct 3D cell-generated substrate deformations of a mesenchymal cancer cell. We show that this algorithm exhibits greatly improved performance relative to existing algorithms, with high recovery ratios for large displacement information, mismatch ratios that are over an order of magnitude smaller, and computational efficiency at least an order of magnitude faster. This performance can be further augmented for large displacements through multi-attribute particles, which encode additional information about each particle. We show a 50% improvement in recovery ratio utilizing two types of particles with different colors, at low and high seeding densities. In principle, this algorithm could also be improved using orthogonal features such as additional colors, particle sizes, shapes, etc.

We envision that T-PT can be generalized more broadly to other physical and biological systems of interest. First, we have demonstrated T-PT for three-dimensional volumes, but it can be utilized similarly for two-dimensional images. Second, T-PT has been used to track particle motion based on two consecutive time steps, but an extension to multi-time point tracking is straightforward. With time-lapse data, multi-frame particle trajectories can increase accuracy by predicting particle positions in successive frames. This information, in conjunction with the iterative deformation warping, reduces the particle search region enhancing the overall tracking performance. Third, our particle tracking scheme can be extended to track any feature or object, if it can be detected and localized within the images. This is also an inherent, and important, limitation of T-PT: it requires particles or other detectable features to be localized within each image for subsequent tracking. In that regard, correlation-based methods are advantageous, as they only need random intensity speckle patterns within the input images. T-PT also makes use of the randomness of the relative particle positions to track the particles and therefore is not optimized for regularly arranged particles or particle lattices. Finally, T-PT is optimized for large numbers of particles, so tracking performance is similar to current SPT techniques for small numbers of particles. It should be noted that T-PT leverages spatial correlations in particle motion to efficiently resolve large displacements. Thus, T-PT enables large performance improvements for directed motion and affine displacements, but can also resolve random and non-affine dynamics.

Single particle tracking algorithms are widely used in biological and physical research and have seen many advances over the past recent years^62^. Nevertheless, existing particle tracking algorithms are challenged by large and complex motion fields with high particle seeding densities. We address this technology gap using feature vectors and an iterative deformation warping scheme, which enable high accuracy in recovering large displacement content, high computational efficacy and the use of multi-attribute particle characteristics. Future microscopy applications may use increasingly large particle numbers. For instance, superresolution microscopy techniques circumvent the diffraction-limited point-spread function, which limited both the spatial resolution of the motion field and the particle seeding density^58^. New structured illumination techniques could conceivably resolve extremely high particle seeding densities with separations below the spatial resolution limit. Simultaneously, increases in camera sensor size could result in unprecedented numbers of particles in the field of view, necessitating high computational efficiency to track the motion field at very high Nyquist spatial frequency. The algorithm demonstrated here is highly promising to meet these emerging technical challenges for ultrahigh resolution light microscopy. Overall, we envision that T-PT will provide the community with a robust, fast and flexible means of tracking complex, two and three dimensional motion fields.

## Methods

### Simulated 3D particle images

The performance of our T-PT algorithm is characterized using a pair of synthetically generated volumetric images of size 512 × 512 × 192 voxels (Supplementary Figure 1). In each reference volume (*t* = *t*_*o*_), unless otherwise stated, 50,000 spherical beads are randomly seeded using a 3D Gaussian intensity profile as an approximation for the optical system’s actual point spread function (PSF). The PSF with amplitude *A* and standard deviation *σ* is expressed as

1 $${\rm{PSF}}(x)=A\,\exp (-\sum _{i=1}^{3}\frac{{x}_{i}^{2}}{2{\sigma }^{2}}).$$

The PSF with a standard deviation *σ* = 1 approximates a spherical particle in the image, with a diameter equal to about 5 voxels (Supplementary Figure 2). To avoid particle overlap in the images, a Poisson disc sampling algorithm is used to seed particles in the images with a minimum separation distance between particles equal to the particle diameter. The particle position in the deformed image is calculated via the imposed displacement field, and the spherical particles are similarly seeded at these positions in the deformed image. To generate the simulated images for each signal-to-noise ratio (SNr), we scale the image intensity such that the peak image intensity is equal to the SNr squared. Then each voxel intensity is replaced by a random number drawn from a Poisson’s distribution with its mean equal to the original intensity.

### T-PT algorithm

#### Particle Detection and Center Localization

The first step in our T-PT algorithm focuses on proper particle detection and center localization using the previously published subpixel-accurate radial symmetry method^39,40^. First, the image is converted into a binary image using a user-defined threshold value, such that only the central region of the particle is segmented as several bright voxels, and the adjacent particle voxels are separated by at least one dark voxel. In the binary image, the simply-connected regions of bright pixels, corresponding to the particle, are computed. Based on the expected particle size, a minimum and maximum number of voxels dependent on the particle size are used to identify connected components of the particles in the image and eliminate noise or multiply-connected particles. This step essentially serves as a user-defined exclusion rule.

Next, an estimate of the particle’s center is constructed from a basic centroid calculation of each of the selected connected components. Then the radial symmetry method is employed using a greyscale image subset around the voxel-level estimate of the particle’s center to compute the actual center position with subpixel accuracy. The particle detection scheme is fully user-configurable to accommodate most commonly used geometric shapes, not just spherical and circular ones.

#### Particle Linking

T-PT is a feature-vector based particle tracking method, which utilizes the randomness of the particle locations to create a particle-descriptor based on the spatial positions of neighboring particles. The particles are linked between two image frames such that each particle pair has the smallest L^2^ particle descriptor distance. The particle descriptor and particle linking process is described in the Results section. To remove false particle links produced during the particle matching step, T-PT utilizes the similarity of neighborhood test and a displacement outlier removal scheme. The similarity of neighborhood test uses the idea that when a particle moves between image frames, it should be surrounded by some of the same neighboring particles. To assure neighborhood similarity, the condition that at least *p* out of *q* nearest neighboring particles are the same from the reference and deformed frames needs to be satisfied. The image dimensionality, displacement field, and particle seeding density dictate appropriate values for *p* and *q*. In our studies, *p* = 2 and *q* = 5 perform well for a variety of displacement fields and particle seeding densities in our 3D images. Additionally, displacements computed from each particle link are analyzed using the universal median test^53^ to remove potential outliers. The links verified by these two tests are converted to successful particle matches.

#### Iterative Deformation Warping

T-PT uses the iterative deformation warping scheme^51^ to track large motion fields as shown in Fig. 1. Let the particle positions in the reference and deformed images in the *k*^*th*^ iteration of deformation warping be ${x}_{i}^{k}$ and ${y}_{i}^{k}$, respectively. From matched particle pairs, the displacement field at matched particle positions in the reference image is calculated as ${u}_{i}^{m}={y}_{i}^{m}-{x}_{i}^{m}$. This displacement field is linearly interpolated to all particle positions *x*_*i*_ and *y*_*i*_ as ${u}_{i}^{k}$ for the *k*^*th*^ iteration of deformation warping. The particle positions in the reference and deformed frames are updated in the *k*^*th*^ + 1 iteration step as

2 $$\begin{array}{ll}{x}_{i}^{k+1} & ={x}_{i}^{k}+\frac{{u}_{i}^{k}}{2}\\ {y}_{i}^{k+1} & ={y}_{i}^{k}-\frac{{u}_{i}^{k}}{2}.\end{array}$$

The particle matching and iterative deformation warping scheme are then iteratively used to track particles on decreasing image subset sizes until the convergence criteria are achieved.

As a final step, the displacements from the tracked particles are interpolated to predict the positions of untracked particles in the reference frame within a user-defined search radius. If a single particle is found within the search radius, and is verified by the earlier outlier removal schemes, the particle pair is added to the list of successful particle matches. This final step improves the recovery ratio of the T-PT algorithm.

#### Implementation

A parallelized version of our T-PT algorithm is implemented in Matlab 2017a, and this algorithm is freely available on our Github page (https://github.com/FranckLab/).

### Hardware

All computations for execution time estimates for the various algorithms were performed on a PC with an Intel i7 6700 K clocked at 4.0 GHz and 32 GB of memory.

### Simulation of non-affine shear deformation

The non-affine shear deformation is generated by applying a homogeneous simple shear deformation of 10% nominal shearing strain along the x-y direction of a volumetric image of size 512 × 512 × 192 voxels as shown in Fig. 4c. Superimposed on the affine displacement field, is a non-affine displacement field of a normal distribution with a mean of zero and a standard deviation of 0.75 voxels along the *x*, *y* and *z* directions. The displacement field was prescribed to the 50,000 particles randomly seeded into synthetically generated images.

### CFD simulation of fluid flow around cylinders

A 2D fluid flow around a stack of periodically-spaced cylinders is simulated using COMSOL Multiphysics 5.2 (Burlington, MA, USA) (Fig. 4f). The computations were performed using the single-phase, time-dependent (unsteady) laminar flow physics module. We use a smoothed step function to ramp up the inlet velocity to 1 m.s^−1^. The fluid is modeled as incompressible with a density of 1 kg.m^−3^ and dynamic viscosity of 1 × 10^−4^ kg.m^−1^.s^−1^. The Eulerian mesh is produced using Comsol’s physics-controlled mesh sequence type with element size set to extremely fine, resulting in a final mesh of 80,638 domain elements and 1078 boundary elements. The fluid displacement field from Comsol model is prescribed to 45,000 particles randomly embedded in a synthetically generated image pair of size 2048 × 2048 pixels.

### Experimental Method

#### Preparation of Polyacrylamide Substrates

Soft polyacrylamide (PA) gels were prepared as previously described by Toyjanova *et al*.^17^. in 24-well glass bottom plates (Cellvis, P24-1.5H-N). Each well was activated via treatment with 0.5% (v/v) of (3-aminopropyl)triethoxysilane (Sigma Aldrich) in ethanol, followed by 0.5% glutaraldehyde (Polysciences, Inc) in deionized water. Hydrophobic cover glass (12 mm diameter, Fisher Scientific) was also prepared by dipping in a solution of 97% (v/v) hexanes (Fisher Scientific), 2.5% (v/v) (tridecafluoro-1,1,2,2-tetrahydrooctyl)-triethoxysilane (Gelest), and 0.5% (v/v) glacial acetic acid (Sigma Aldrich), which was allowed to dry at room temperature. Next, a PA gel solution was prepared using 3% acrylamide (40% w/v, BioRad) and 0.2% N,N-methylene-bisacrylamide (2% w/v, BioRad) to yield a gel with final stiffness of ~1.5 kPa^17^. Two different color fluorescent beads were also incorporated into the PA gel solution to improve tracking by using multi-attribute particles for T-PT. Carboxylate modified microspheres (FluoSpheres) were added at a final concentration of 14% and 2%, for 1.0 μm diameter red fluorescent (580/605 nm) and 2.0 μm diameter blue-green fluorescent (430/465 nm) beads, respectively. We used beads of different sizes due to the lack of availability of both color beads at 1.0 μm diameter. In experiments, we utilized only the color to differentiate between the beads. Next, 12 μL of PA gel solution was pipetted into the activated glass well and flattened with the hydrophobic round cover glass to yield a PA gel of ~70 μm in thickness. The PA solution was allowed to completely gel (30 minutes) before removal of the hydrophobic coverslip. Gels were kept hydrated in phosphate buffered saline (1X PBS) prior to functionalization for cell studies.

#### Functionalization of Polyacrylamide Substrates for Cell Adhesion

To facilitate cell adhesion to PA gels, the bifunctional crosslinker sulfo-SANPAH was deposited onto the gel surface, enabling subsequent collagen I conjugation. PBS was removed from the PA gels in 24-well plates and a solution of 1 mg.mL^−1^ sulfo-SANPAH (ThermoFisher Scientific) in deionized water was added on top of each gel to yield 25 μg.cm^−2^ sulfo-SANPAH. Next, the plate was exposed to UV light for 15 minutes for photoactivation of the crosslinker, the darkened sulfo-SANPAH solution was removed, and the process was repeated for a second coating of sulfo-SANPAH. After the second deposition, sulfo-SANPAH was removed and the wells were washed several times in 1X PBS to remove unreacted sulfo-SANPAH. PA gels were then incubated with a dilute solution of rat tail collagen I (Fisher Scientific) in 0.02 N acetic acid overnight at 4°C, to yield a final protein coating of 5 μg.cm^−2^. The next day, PA gels were washed twice with 1X PBS to remove residual collagen I and acetic acid.

#### 3D Traction Force Microscopy with Breast Cancer Cells

Human metastatic breast adenocarcinoma cells (MDA-MB-231) were used for TFM studies. MDA-MB-231 cells with cytoplasmic green-fluorescent protein (GFP) expression were a generous gift from S. Javaid and D. Haber from the Massachusetts General Hospital. Cells were cultured in growth media containing Dulbecco’s Modified Eagle’s medium (DMEM) with L-glutamine, 4.5 g.L^−1^ glucose, sodium pyruvate (Fisher Scientific, MT-10-013-CV) and supplemented with 10% fetal bovine serum and 1% penicillin/streptomycin (Fisher Scientific). Cells were maintained at 37°C and 5% CO_2_ in a humidified incubator. The 24-well plates with collagen I coated soft PA gels were equilibrated with 500 μL MDA-MB-231 growth medium for 30 minutes at 37 °C. Media was aspirated from the wells and MDA-MB-231 GFP-cytoplasm cells were seeded onto PA gels at a density of 3,125 cells cm^−2^ in a total volume of 1 mL of media per well. Cells were allowed to settle and adhere overnight prior to time-lapse fluorescence microscopy. Cells and PA gels were imaged using a Nikon AR-1 confocal system mounted on a Ti-Eclipse inverted optical microscope controlled by NIS-Elements Nikon Software with a S Plan Fluor ELWD 20 × Ph1 ADM objective (NA = 0.45; Nikon). The blue beads, cells and red beads were excited using lasers of wavelength 405 nm, 488 nm and 561 nm, respectively. A confocal image stack of 512 × 512 × 101 voxels (321.9 × 321.9 × 60.0 μm^3^) with a z-step of 0.60 μm was recorded every 30 minutes for 2 hours, and cells were maintained at 37°C throughout the experiment via an incubated microscope chamber and thermocouple. After the final time-point of live imaging, media was aspirated, 1X PBS was added to wash the wells, and 0.05% Trypsin (Fisher Scientific) was added to dissociate cell-matrix attachments. Once cells were fully detached from the PA gels, a final z-stack was acquired as a stress-free reference image for comparison to cell-based PA gel deformation and extraction of TFM measurements.

## Electronic supplementary material

Supplementary Information
