# Supplementary material for: Rapid, topology-based particle tracking for high-resolution measurements of large complex 3D motion fields
Source: Sci Rep. 2018 Apr 3;8:5581. doi: 10.1038/s41598-018-23488-y (PMC5882970; doi:10.1038/s41598-018-23488-y)
Supplement: Supplementary file 1 — Supplementary Information [file 41598_2018_23488_MOESM1_ESM.pdf]

Supplementary Information: Rapid, topology-based particle tracking for high-resolution measurements of large complex 3D motion fields

Mohak Patel[1], Susan E. Leggett[1,2,3], Alexander K. Landauer[1], Ian Y. Wong[1,2,3] & Christian Franck[1,3]

1. School of Engineering, Brown University, Providence, RI 02912, USA
2. Pathobiology Graduate Program, Brown University, Providence, RI 02912, USA
3. Center for Biomedical Engineering, Brown University, Providence, RI 02912, USA

Table of Content

**Supplementary Note 1**	Particle volumetric images	2
**Supplementary Note 2**	Nyquist resolution calculation	2
**Supplementary Note 3**	Mean displacement error versus signal-to-noise ratio	3
**Supplementary Note 4**	T-PT performance for non-affine motion in shear deformation	3
**Supplementary Note 5**	Random particle motion	4
**Supplementary Note 6**	Time-lapse particle motion with particles randomly disappearing and appearing	5
**Supplementary Note 7**	T-PT algorithm workflow	7
**Supplementary Figure 1**	Synthetically generated 3D volume of size 512x512x192 voxels consisting of 50,000 spherical particles.	2
**Supplementary Figure 2**	Synthetic image of a single simulated spherical particle.	2
**Supplementary Figure 3**	Mean displacement error of T-PT versus a range of SNr values.	3
**Supplementary Figure 4**	Tracking performance of T-PT for an applied simple shear deformation with a varying level of non-affine deformation.	4
**Supplementary Figure 5**	Performance of various SPT algorithms in tracking random particle motion.	5
**Supplementary Figure 6**	Performance characterization of T-PT in tracking particles across time-lapse image series where particles randomly disappear and appear between image frames.	6
**Supplementary Figure 7**	Flowchart showing major execution steps of the T-PT algorithm.	7

1. Particle volumetric images

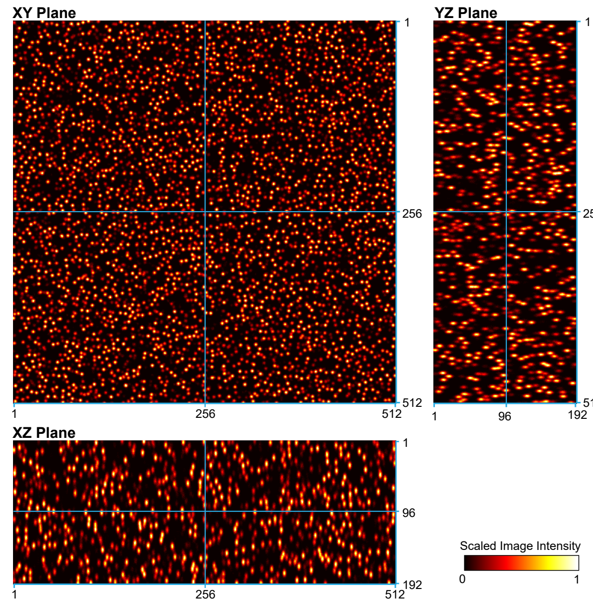

Figure 1: A simulated 3D volume of size 512x512x192 voxels consisting of 50,000 randomly distributed spherical beads.

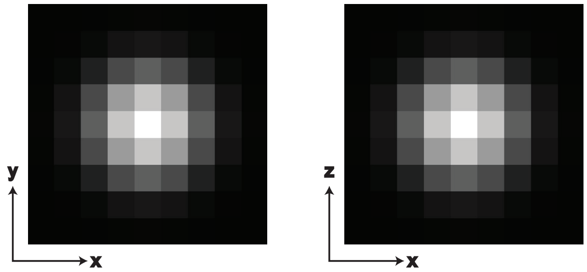

Figure 2: A synthetic image of a single simulated spherical particle.

2. Nyquist resolution calculation

For a given particle seeding density in the image, ρ_o, the mean particle distance in the images is,

$$r_{2D} = \sqrt{\frac{1}{\pi \rho_{o,2D}}},$$

$$r_{3D} = \sqrt[3]{\frac{3}{4\pi\rho_{o,3D}}},$$

for 2D and 3D images respectively[1]. Here, $\rho_{o,2D}$ is the particle seeding density in the 2D image defined as the number of particles per unit image size and $\rho_{0,3D}$ is the particle seeding density in the 3D image defined as the number of particles per unit 3D image volume.

According to the Nyquist-Shannon sampling theorem, the spatial resolution of the recovered displacement field is determined by half the sampling frequency. Therefore for a given mean particle distance, r, a first order estimate for the Nyquist spatial resolution of the recovered displacement field is $2r$.

3. Mean displacement error versus signal-to-noise ratio

In Supplementary Figure 3, we plot the mean displacement error for our T-PT technique against synthetically generated images across a range of Poisson-distributed noise to account for variable experimental settings. The mean displacement error is defined as the mean of the difference between the imposed versus the measured particle displacement vector magnitude. The plot demonstrates that T-PT has low tracking error even for images with relatively high noise levels.

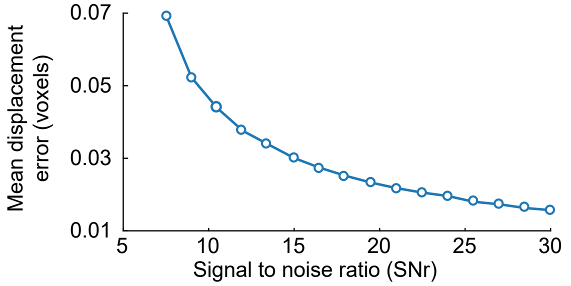

Figure 3: The mean displacement error of T-PT calculated from tracking particles using synthetic images with a range of SNr values.

4. T-PT performance for non-affine motion in shear deformation

In the results section, we show the ability of T-PT to recover non-affine shear deformations. Here, we showcase tracking performance for the same shear deformation but for a range of different non-affinity. To define non-affinity in the images, we introduce a non-affinity parameter, ND, defined as the ratio of maximum non-affine displacement magnitude to the mean particle separation distance. As in the main text example, the non-affine shear deformation is generated by applying a homogeneous simple shear deformation of 10% nominal shearing strain along the $x - y$ direction of a volumetric image of size 512x512x192 voxels. Superimposed on the affine displacement field is a non-affine displacement field of a truncated zero-mean normal distribution to two standard deviations in a range of standard deviations along the x, y and z directions. The displacement field

was prescribed to the 50,000 particles randomly embedded in the synthetically generated images. T-PT is used to track this non-affine shear deformation from these synthetically generated images. In Supplementary Figure 4, we plot the recovery and mismatch ratio for T-PT for a range of non-affinity in the particle motion field, which shows that T-PT can accurately recover very large non-affine shear deformations.

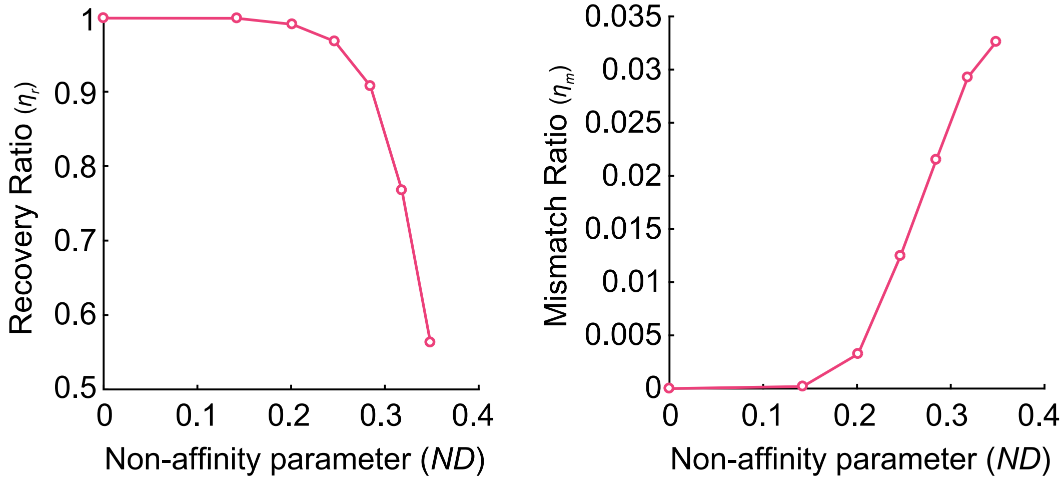

Figure 4: Tracking performance of T-PT for an applied simple shear deformation with a varying levels of non-affine deformations. Plot of recovery and mismatch ratio for a range of the non-affinity parameter (ND).

5. Random particle motion

The tracking performance of T-PT for random particle motion was evaluated by simulating random particle motion in 3D images. We synthetically generated a 3D volume of 512x512x192 voxels seeded with 50,000 identically-sized spherical particles with an SNr of 25. The particles were prescribed with a random displacement vectors of a truncated zero-mean normal distribution to two standard deviations in a range of standard deviations along the x, y and z directions. We evaluated the performance of T-PT and other current SPT techniques as a function of increasing displacement parameter d for the prescribed displacement field (Supplementary Figure 5). For $d < 0.25$, T-PT and TrackMate exhibit very high recovery ratios $\eta_r \sim 1$. As d was increased further, the η_r of TrackMate remains close to unity out to $d \sim 0.4$, while T-PT decreases to $\eta_r \sim 0.8$. In comparison, Legant et al.'s method maintains a stable $\eta_r \sim 0.97$ until $d \sim 0.3$, but with increasing d, recovery ratio decreases to $\eta_r \sim 0.5$. FVRM starts with a lower $\eta_r \sim 0.9$, which decreases with increasing d (Supplementary Figure 5).

For $d < 0.25$, T-PT, TrackMate and Legant et al.'s method all have very low mismatch ratios $\eta_m \sim 0$. For increasing d, T-PT's mismatch ratio increases to $\eta_m \sim 0.02$, in comparison the η_m of TrackMate and Legant et al.'s method increases at a lower rate. FVRM has the highest η_m,

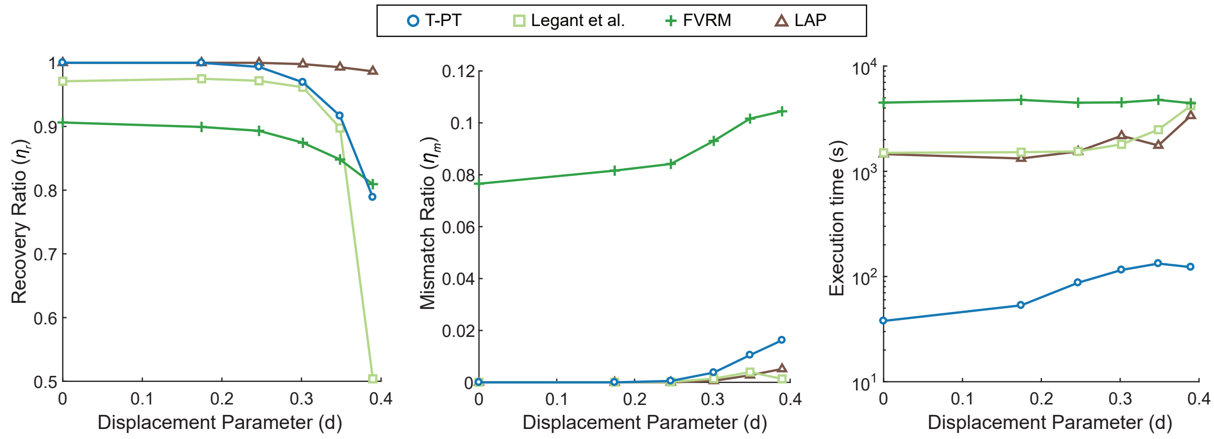

Figure 5: Performance charactertization of various SPT algorithms in tracking random particle motion. Plots show recovery ratio, mismatch ratio and execution time versus increasing displacement parameter. Here, 'T-PT' indicates the topology-based particle tracking method introduced here; 'Legant et al.' indicates the feature-vector based particle tracking method by Legant et al.[2, 3]; 'FVRM' indicates the feature-vector based relaxation method[4]; 'LAP' indicates Jaqaman et al.'s LAP-based algorithm [5] implemented in TrackMate [6].

which increases with increasing d. We also compare the execution time of all the SPT algorithms for increasing d. The execution time of T-PT, TrackMate, and FVRM increase with increasing d, while FVRM has the slowest execution time and it remains at a nearly constant value for increasing d. T-PT tracked particles in 122.7 seconds for $d \sim 0.4$, which is 27x faster than TrackMate, the second fastest method. For all d, T-PT is faster by at least an order of magnitude than the second fastest method.

These results show that TrackMate, followed by T-PT, enables the most accurate tracking of particles undergoing random motion. However, the fast execution time of T-PT can make it preferable even for certain purely random motion applications, especially those involving small random particle motion at very high particle numbers.

6. Time-lapse particle motion with particles randomly disappearing and appearing

We evaluate the performance of T-PT to track particle motion in a time-lapse image series in which particles randomly disappear and appear between image frames. By employing synthetic images we mimicked experimental particle tracking conditions in which particles can disappear, due to moving out of the image frame, detection failure or particle merging, or in which particles can appear due to moving into the image frame, failure of particle detection in the previous image frame or particle splitting. In the simulations, identical spherical particles were seeded in a space large enough such that the final image of 512x512x192 voxels region used as input for the algorithm was fully seeded with approximately 50,000 particles throughout the application of the motion field. We generated a time-lapse image series for six time points. The particles were prescribed

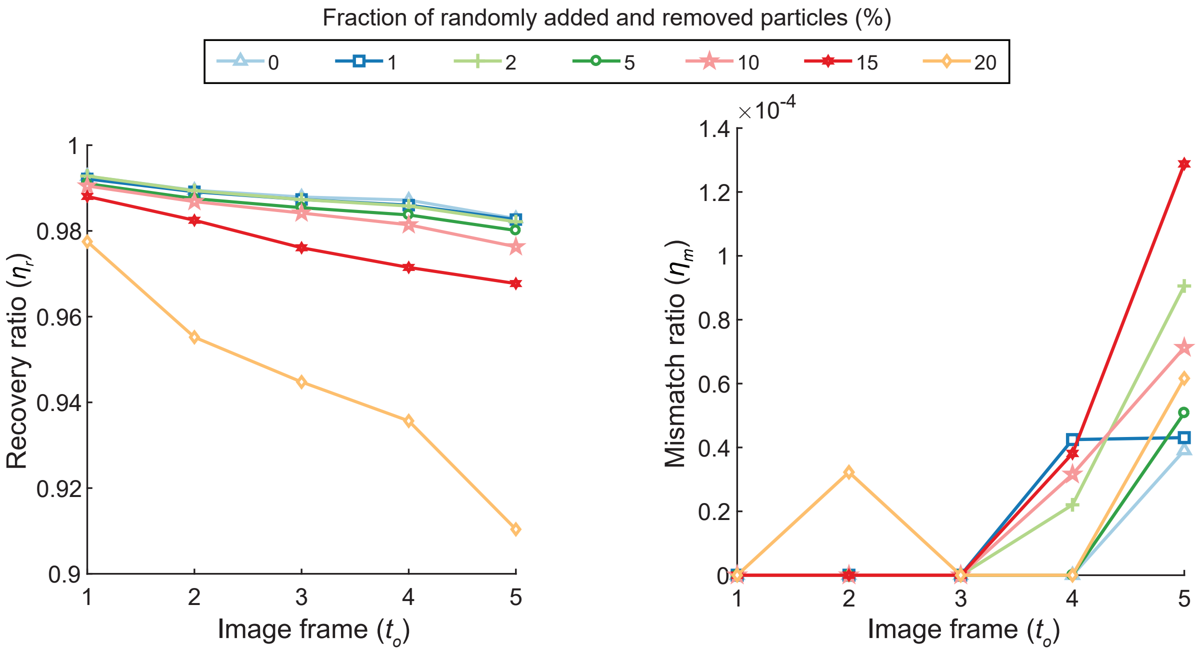

Figure 6: Performance characterization of T-PT in tracking particles across time-lapse image series were particles randomly disappear and appear between image frames. The plots of recovery and mismatch ratio from the initial image frame ($t = 0$) to a current image frame ($t = t_0$) against progressive image frames for a varying fraction of particles randomly added and removed between image frames are shown.

the displacement field from Figure 2a, with a displacement parameter d of 1 between each time frame. Between each time point, about 1250 particles (2.5%) move out of the image frame and a similar number of new particles enter the image frame due to the underlying particle motion field. Additionally, we randomly seeded and removed a varying fraction of particles in each frame to simulate particles appearing and disappearing in experimental multi-time point particle tracking. The number of particles seeded and removed in each image frame was picked from a Gaussian distribution having a mean equal to the number of particles intended to be seeded and removed with a standard deviation of 20% of the mean value.

To track particles across the time-lapse data, T-PT matches particles between two consecutive image frames. The particle trajectories across multiple frames are later merely computed from joining the frame-to-frame particle tracking results. In Supplementary Figure 6, we plot the recovery and mismatch ratios of the particles tracked between the initial image frame ($t = 0$) to the current image frame ($t = t_o$). The recovery ratio of the algorithm decreases with increasing image frame. This is expected because T-PT computes particle trajectories from consecutive frame-to-frame particle matches without employing any gap closing schemes. As a result, the recovery ratio is compounded over image frames. Moreover, the recovery ratio of the algorithm decreases with an increase in the fraction of seeded/removed particles. The algorithm maintains a very high $\eta_r \sim 0.98$

until the 5th image frame for 10% of particles seeded/removed. The recovery ratio at the 5th image frame drops to $\eta_r \sim 0.91$ for 20% of seeded/removed particles for each image frame. The mismatch ratio for all the cases is very low, $\eta_m < 1.4 \times 10^{-4}$. Overall, these results demonstrate that T-PT can accurately track particles across time-lapse data even when a significant fraction of particles disappear and appear between image frames. These results could be further improved using gap-closing schemes.

7. T-PT algorithm Workflow

In this section we describe the workflow of the T-PT algorithm (Supplementary Figure 7). In particular, we explain each step in the algorithm and the effect of various parameters on the algorithm performance.

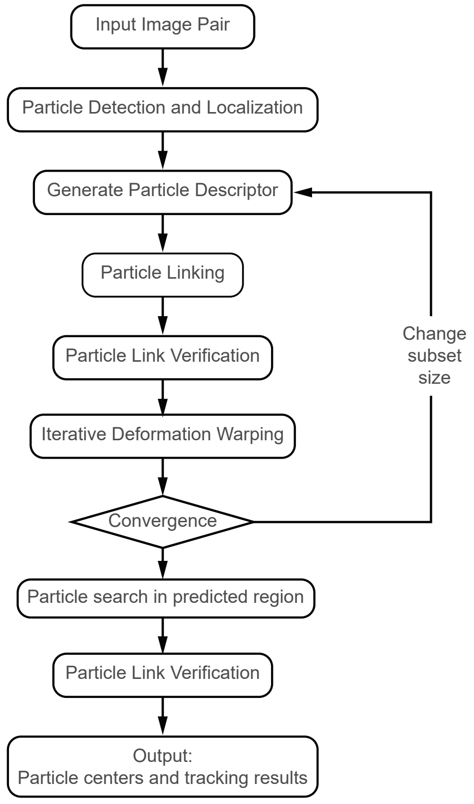

Figure 7: Flowchart showing the major execution steps of the T-PT algorithm.

1. *Input image pair:*

   T-PT loads a consecutive image pair for every time point in the time-lapse image series. Each time point image is stored within a cell container 'vol' inside a binary .mat file (the standard file extension to store MATLAB formatted data).

2. *Particle detection and localization:*

   First, T-PT normalizes the input images such that a voxel has a maximum intensity value of 1. To detect particles in the images, T-PT converts the input images into binary images using image thresholding operations. Image thresholding operations are used to segment the central particle region as bright voxels and background as dark voxels. The default image thresholding value is 0.5. However, the user should set the image thresholding value such that adjacent particles are separated by at least one dark voxel.

   From the binary images, the algorithm computes simply-connected regions of bright voxels. To eliminate simply-connected regions corresponding to noise or multiply connected particles, the user needs to define the minimum and maximum voxel size for the simply-connected region of the particle based on its size within the image. The default value for the minimum and the maximum number of voxels is 4 and infinity, respectively. The algorithm computes the centroid of each filtered simply-connected region to estimate a voxel-level particle center.

   The radial symmetry method [7, 8] computes each particle center with sub-voxel accuracy from the original image subset surrounding each estimated particle center. The user needs to set the image subset size such that each particle just fits inside the image subset. The default image subset size is 7x7x7 voxels.

3. *Generating the particle descriptor:*

   As described in the Results section, T-PT computes the particle descriptor for each particle in the reference and deformed image. By default, the algorithm uses 16 nearest neighboring particles, n, and two concentric shells, k, to create the particle descriptor. The reported default values of n and k have been found empirically to work well for a variety of motion fields and particle seeding densities in 3D images. While changing values of n and k do not significantly affect the algorithm's performance, the users can optimize these parameters for the motion fields within their own images.

   For small n, the particle descriptor bins get sparsely filled and thus reduce the uniqueness of particle descriptor. For large n, each particle descriptor bin gets filled with many particles. Since the particles are randomly distributed, each bin in the particle descriptor gets a similar number of particles, and thus reduces the uniqueness of the particle descriptor.

   Tests on simulated motion fields showed that $k = 1$ only allowed for eight bins in the particle descriptor. The length of this particle descriptor is too short in most cases to create a unique particle descriptor signature for a large number of particles. For large k, the particle descriptor bin size reduces to a small volume, which does not allow particles enough room to move without significantly altering the particle descriptor.

4. *Particle linking:*

   The reference and deformed volumes are divided into cubic volume subsets for particle linking. The particles from the reference image subset are linked to the corresponding deformed image subset. To allow particles near the edge of the reference image subset to undergo large displacement while still being in the same corresponding deformed image subset, the deformed

image subset size is always set to be twice the size of the reference image subset. However, the reference and deformed image have the same centers. The initial subset size should be larger than the maximum particle motion in the images. The default value of the initial subset size is 256 voxels. In the iterative particle matching process, the size of the image subset remains the same or decreases by a factor of two, until the convergence criteria are met.

The particles are linked between the reference image subsets and the corresponding deformed image subsets such that a chosen particle pair has the smallest L^2 particle descriptor distance. Assuming, r and s to be a particle descriptor for a particle in the reference and deformed images respectively, the L^2 particle descriptor distance between r and s is defined as,

$$L^2 = \sqrt{\sum_{i=1}^{8k} (r_i - s_i)^2}. \tag{1}$$

Here, r and s are expressed as a vector having $8k$ (number of bins) components. Then, the algorithm eliminates any particle links which are not bijective to enforce a one-to-one particle linking condition.

5. *Particle link verification:*

   T-PT uses the similarity of neighborhood test and the universal median filter test as outlier removal schemes to remove spurious particle links. For the similarity of neighborhood test, the default values of $p = 2$ and $q = 5$ are empirically found to work well for a variety of motion fields and particle seeding densities in our 3D images. A small p/q ratio relaxes the outlier removal criterion for the similarity of neighborhood test, which is suited for high spatial frequency motion as the local relative particle positions change vastly. A high p/q ratio is appropriate for low spatial frequency motion as the the local relative particle position does not change significantly.

   In the universal median test [9], 27 neighbors are used to compute the residual from the displacement for each particle link. The residual is normalized by the median residual of these 27 neighboring particles. Any particle link exceeding the normalized residual value above a user-defined threshold value is removed. By default, the residual threshold value is 4. Increasing the threshold value relaxes the outlier removal criteria and is suited for very high spatial frequency motion fields while a low threshold value is appropriate for a low spatial frequency motion field.

   Overall, relaxing the outlier removal criteria increases the number of particle matches found by the algorithm, but it can also lead to a higher number of spurious particle matches. Whereas setting stricter outlier removal criteria will decrease the number of particle matches found by the algorithm, but it also reduces the number of false particle matches. The user can optimize the extent of the outlier removal process to level appropriate to their application and motion field.

6. *Iterative deformation warping:*

Iterative deformation warping is used to warp particle positions in reference and deformed image volumes based on cumulative particle matches. IDM helps in the recovery of large particle displacements, and improves the similarity of the particle descriptor in the reference and deformed volumes, which further enhances the accuracy of the particle linking process. Assume x_i^k and y_i^k to be the particle positions in the reference and deformed images in the kth iteration of deformation warping. From matched particle pairs, the displacement field at matched particle positions in the reference image is calculated as $u_i^m = y_i^m - x_i^m$. The displacement field is linearly interpolated to all particle positions x_i and y_i as u_i^k for the kth iteration of deformation warping. The particle positions in the reference and deformed frame are updated in the $k^{th}+1$ iteration step as,

$$\begin{aligned} x_i^{k+1} &= x_i^k + \frac{u_i^k}{2} \\ y_i^{k+1} &= y_i^k - \frac{u_i^k}{2}. \end{aligned} \tag{2}$$

7. *Convergence criteria:*

In each iteration step of the iterative particle matching and IDM process, T-PT computes the total fraction of particles matched between the image pair. After each iteration, the fraction of particle matches increases. The algorithm meets the convergence criteria when the fraction of matched particles after each iteration reaches a steady value. Specifically, the convergence criteria is reached if the current subset size is 16 voxels and if any of the following conditions are met:

- The algorithm has run through a maximum of 5 iterations for a subset size of 16 voxels.
- The fraction of matched particles has improved from the previous iteration by an amount smaller than 0.25%.
- The ratio of the fraction of matched particles between the current and previous iterations is less than 0.1.

If the convergence criteria are not met, the algorithm undergoes another iteration of particle matching and IDM. In the next iteration of particle matching, the subset size reduces by a factor of 2 if any of the following conditions are achieved. Otherwise, the subset size for the next iteration remains the same.

- The algorithm has run through a maximum of 5 iterations for the same current subset size.
- The fraction of matched particles has improved from the previous iteration by an amount smaller than 4%.
- The ratio of the fraction of matched particles between the current iteration and the previous iteration is less than 0.6.

The minimum subset size is limited to 16 voxels. If the algorithm tries to set the subset size below 16 voxels, it is reset back to 16 voxels, unless the user specifies a smaller subset size.

8. *Particle search in predicted regions:*

   As a final step to improve the recovery ratio of the algorithm, displacements from the matched particles are interpolated to predict the position of unmatched particles in the deformed volume with a default search radius of one-third of the nearest neighbor particle separation distance to the unmatched particle in the reference image. If the algorithm finds a single particle in the predicted region in the deformed image, it saves the particle pair as a temporary particle link.

9. *Particle link verification:*

   The temporary particle links formed in the previous step are subjected to the earlier described outlier removal scheme comprised of the similarity of neighborhood test and the universal median test [9]. The links verified by the outlier removal schemes are stored in the list of successful particle matches between the image pair.

10. *Output:*

    The particle centers from the reference and deformed images and the particle matches are saved as the output of the algorithm.

References

[1] Pereira, F., Stüer, H., Graff, E. C. & Gharib, M. Two-frame 3d particle tracking. *Measurement Science and Technology* **17**, 1680 (2006).

[2] Legant, W. R. *et al.* Measurement of mechanical tractions exerted by cells in three-dimensional matrices. *Nature Methods* **7**, 969–971 (2010).

[3] Legant, W. R. *et al.* Multidimensional traction force microscopy reveals out-of-plane rotational moments about focal adhesions. *Proceedings of the National Academy of Sciences* **110**, 881–886 (2013).

[4] Feng, X., Hall, M. S., Wu, M. & Hui, C.-Y. An adaptive algorithm for tracking 3d bead displacements: application in biological experiments. *Measurement Science and Technology* **25**, 055701 (2014).

[5] Jaqaman, K. *et al.* Robust single-particle tracking in live-cell time-lapse sequences. *Nature Methods* **5**, 695–702 (2008).

[6] Tinevez, J.-Y. *et al.* Trackmate: An open and extensible platform for single-particle tracking. *Methods* **115**, 80–90 (2017).

[7] Parthasarathy, R. Rapid, accurate particle tracking by calculation of radial symmetry centers. *Nature Methods* **9**, 724–726 (2012).

[8] Liu, S.-L. *et al.* Fast and high-accuracy localization for three-dimensional single-particle tracking. *Scientific Reports* **3** (2013).

[9] Westerweel, J. & Scarano, F. Universal outlier detection for piv data. *Experiments in Fluids* **39**, 1096–1100 (2005).
